# Identification of key pharmacodynamic markers of American ginseng against heart failure based on metabolomics and zebrafish model

**DOI:** 10.3389/fphar.2022.909084

**Published:** 2022-10-14

**Authors:** Rong Dong, Yougang Zhang, Shanjun Chen, Huan Wang, Kaiqing Hu, Huanxin Zhao, Qingping Tian, Kewu Zeng, Songsong Wang, Liwen Han

**Affiliations:** ^1^ School of Pharmacy and Pharmaceutical Science, Shandong First Medical University and Shandong Academy of Medical Sciences, Jinan, China; ^2^ School of Pharmaceutical Science of Shanxi Medical University, Taiyuan, China; ^3^ School of Pharmaceutical Science of Peking University, Beijing, China

**Keywords:** American ginseng (Panax quinquefolius L.), anti-heart failure, metabolomics, pharmacodynamic markers, zebrafish, network pharmacology

## Abstract

**Background:** American ginseng (*Panax quinquefolium* L., AG) is a traditional Chinese medicine with multiple cardiovascular protective properties. Many bioactive components have been discovered in AG over these years. However, the understanding of these key pharmacodynamic components of activity against heart failure is insufficient.

**Methods:** A heart failure model was established using AB line wild-type zebrafish (*Danio rerio*) to evaluate the anti-heart failure activity of AG. Untargeted metabolomics analysis based on ultra-high performance liquid chromatography-quadrupole electrostatic field orbitrap-mass spectrometry technology (UHPLC-QE-Orbitrap-MS) was performed to screen differential components from AG samples. The potential active components were verified using the zebrafish model. Simultaneously, network pharmacology and molecular docking techniques were used to predict the possible mechanism. Finally, the key targets of six key pharmacodynamic components were verified in zebrafish using quantitative real-time-polymerase chain reaction (Q-PCR) techniques.

**Results:** The heart failure model was successfully established in 48 h of post-fertilization (hpf) zebrafish larvae by treating with verapamil hydrochloride. The zebrafish assay showed that the anti-heart failure effects of AG varied with producing regions. The result of the herbal metabolomic analysis based on UHPLC-QE-Orbitrap-MS indicated that ginsenoside Rg3, ginsenoside Rg5, ginsenoside Rg6, malic acid, quinic acid, L-argininosuccinic acid, 3-methyl-3-butenyl-apinosyl (1→6) glucoside, pseudoginsenoside F11, and annonaine were differential components, which might be responsible for variation in efficacy. Further analysis using zebrafish models, network pharmacology, and Q-PCR techniques showed that ginsenoside Rg3, ginsenoside Rg5, ginsenoside Rg6, malic acid, quinic acid, and pseudoginsenoside F11 were the pharmacodynamic markers (P-markers) responsible for anti-heart failure.

**Conclusion:** We have rapidly identified the P-markers against heart failure in AG using the zebrafish model and metabolomics technology. These P-markers may provide new reference standards for quality control and new drug development of AG.

## 1 Introduction

Panacis Quinquefolii Radix, also known as American ginseng (AG), is the dry root of *Panax quinquefolium* L, belonging to the Araliaceae family (Huang et al., 2019). AG has been used in protecting cardiovascular lesions (Szczuka et al., 2019), glycemic control (Vuksan et al., 2019), delaying neurodegenerative diseases (Xie et al., 2018), regulating immune systems (Ghosh et al., 2020), and inhibiting tumor and infection (L. Qi et al., 2010; L. Wang et al., 2020) for a significant time. Ginsenosides were considered one major kind of active components in AG (Yuan et al., 2010). Many research studies have reported that ginsenosides increased ventricular myocyte viability, alleviated the early apoptosis of ventricular myocytes, and promoted energy production *via* the activation of AMP-activated protein kinase (AMPK) metabolic pathways (Yu et al., 2021). AG from North America was verified for its ability to attenuate β-adrenergic activation-induced heart failure *via* the prevention of protein kinase A activation and inhibition of cyclic AMP response element-binding protein phosphorylation (Tang et al., 2016). However, these known active compounds do not seem capable of explaining the anti-heart failure response of AG; hence, it is necessary to explore the more active components in AG.

Traditional Chinese medicine (TCM) is a relatively complex chemical composition system. Hence, exploring the relationship between active components and diseases is difficult (Zhang R et al., 2019). Metabolomics is a high-throughput, high-sensitivity, and unbiased approach (Rinschen et al., 2019), offering us a powerful technology for analysis of complex TCM. Over the years, metabolomics technology has been widely used for quality control of TCM (H. Xiong et al., 2020), pharmacodynamics analysis (Y. P. Shi et al., 2020), and toxicity evaluation (Duan et al., 2018). Especially, some innovative concepts based on metabolomics were proposed for exploring the scientific connotation of TCM, such as chinmedomics (Zhang A. H et al., 2019), functional metabolomics (T. Wang et al., 2021), and spatial metabolomics (Nakabayashi et al., 2021).

Zebrafish (*Danio rerio*) is an international recognized vertebrate model possessing various characteristics, such as fast reproduction, low consumption, and a short experimental period (MacRae and Peterson, 2015). The zebrafish genome is genetically similar to the human genome. Also, zebrafish has been used for high-throughput drug screening and drug toxicity evaluation (Horzmann and Freeman, 2018). Heart failure is defined as the heart’s inability to supply enough blood to meet the body’s needs (Pfeffer et al., 2019). The heart structure, physiological functions, signal pathways, and ion channels of zebrafish are highly comparable with those of humans (X. Shi et al., 2018). In comparison with the mammalian models, heart failure models in zebrafish are simple, low-cost, and beneficial for mechanistic studies. Also, the embryonic cardiac function is easy to quantify and observe (Narumanchi et al., 2021). When injured, zebrafish can rapidly and effectively regenerate their hearts. Several zebrafish models of heart failure were established by genetic manipulation or by drug treatment. Many drugs, including isoproterenol (Wang J et al., 2019), aristolochic acid (X. Shi et al., 2017), and verapamil (X. Y. Zhu et al., 2018), could induce heart failure in zebrafish. Hence, zebrafish is an excellent animal model for studying heart-related diseases.

Therefore, in this study, we aimed to discover key anti-heart failure active components and pharmacodynamic markers (P-markers) in AG using metabolomics technology and the zebrafish model. Also, network prediction of the mechanism of AG against heart failure was achieved *via* network pharmacology, molecular docking techniques, and Q-PCR techniques. We hope these P-markers might play an important role in the qualitative evaluation of AG in the future.

## 2 Materials and methods

### 2.1 Chemicals, reagents, and instruments

Pronase E (Lot No. 41844523), tricaine, and N-phenylthiourea (Lot No. BCCC9256) were purchased from Sigma-Aldrich Trading Co., Ltd. (Shanghai, China). Dimethyl sulfoxide (DMSO, Lot No. K1723046), verapamil hydrochloride (Lot No. A2007039), and ammonium bicarbonate (analytical grade) were acquired from Aladdin Co. Digoxin (DIG, Lot No. XNPO-TP) was supplied by Tokyo Chemical Industry. Trichloromethane, propan-2-ol, ethanol, and analytical-grade formic acid was acquired from Sinopharm Chemical Reagent Co., Ltd. (Shanghai, China). HPLC-grade acetonitrile was purchased from Oceanpak (Goteborg, Sweden). HPLC-grade methanol was purchased from Fisher. Ultrapure water was supplied by Watsons Ltd., (Hong Kong, China). Reference standard ginsenoside Rg3 (G-Rg3, Lot No. AF8091509) was purchased from Chengdu Alfa Biotechnology Co., Ltd. Ginsenoside Rg5 (G-Rg5, Lot No. B01SS11S122325), ginsenoside Rg6 (G-Rg6, Lot No. Y8D1H34968), malic acid (MA, Lot No. Z29O10H101559), quinic acid (QA, Lot No. A06N11L130218), and L-argininosuccinic acid (AS, Lot No. SLCC7592) were acquired from Sigma-Aldrich (Shanghai) Trading Co., Ltd. Pseudoginsenoside F11 (Lot No. KA0812CA14) was provided by Shanghai Yuanye Bio-Technology Co., Ltd. The purity of all chemicals was > 98%.

AG samples and their collection times are listed in Table 1. All AG samples were identified as the dry root of *Panacis quinquefolium* L. by Dr. Liwen Han. All samples were stored at the School of Pharmacy and Pharmaceutical Science, Shandong First Medical University.

**TABLE 1:** Collection information of AG samples.

No.	Producing area	Collected time	No.	Producing area	Collected time
S1	Wendeng, Shandong	September 2020	S11	Jilin	August 2019
S2	Wendeng, Shandong	September 2020	S12	Liuba, Shanxi	January 2020
S3	Wendeng, Shandong	September 2020	S13	Xinbin, Liaoning	January 2020
S4	Wendeng, Shandong	September 2020	S14	Wisconsin, United States	November 2016
S5	Wendeng, Shandong	September 2020	S15	Canada	September 2019
S6	Wendeng, Shandong	September 2020	S21	Xinbin, Liaoning	November 2019
S7	Wendeng, Shandong	September 2019	S22	Xinbin, Liaoning	December 2019
S8	Beijing	January 2020	S23	Xinbin, Liaoning	January 2020
S9	Changbaishan, Jilin	September 2019	S24	Xinbin, Liaoning	November 2020
S10	Changbaishan, Jilin	September 2019			

### 2.2 Zebrafish husbandry and embryo collection

The wild AB line zebrafish were provided by the Zebrafish Research Center of the School of Pharmacy and Pharmaceutical Science, Shandong First Medical University and were cultivated in a professional breeding system (Shanghai Haisheng Biological Experimental Equipment Co., Ltd.). Adult zebrafish were reared in a semi-static system at 28°C under a 14-h light/10 h dark cycle (Progatzky et al., 2019) and fed with artemia twice a day. Before the night, adult male and female zebrafish were placed in a mating tank, at a ratio of 2:2. The next day, the zebrafish were stimulated by light to mate and spawn naturally. The embryos were collected within 1 h of spawning and washed with fish water. The clean embryos were transferred into the E3 medium (5 mM NaCl, 0.17 mM KCl, 0.4 mM CaCl_2_, and 0.16 mM MgSO_4_) (H. Sun et al., 2019) and placed in a light incubator at 28.5°C for subsequent experiments. After 24 h, 0.2 mM N-phenylthiourea was added to the E3 medium to prevent the embryos from forming melanin. The animal study was reviewed and approved by the Ethics Committee of Shandong First Medical University and Shandong Academy of Medical Sciences.

### 2.3 Preparation of sample solutions

The dried AG was pulverized and sifted through a 50-mesh sieve. Then, 40 g of AG powder was extracted with 400 ml water twice by refluxing for 2 h. The two filtrates were combined and concentrated at 200 ml in a rotary evaporator. Then, four times the amount of ethanol was slowly added with constant stirring. After standing at 4°C for 24 h, the supernatant was collected *via* centrifugation and concentrated to a certain volume in a rotary evaporator. Finally, the sample was dried in a vacuum drying oven to obtain the AG extract. The tricaine solution (1 mg mL^−1^) was prepared with distilled water. Verapamil hydrochloride was made to 100 mg mL^−1^ with dimethyl sulfoxide (DMSO). Digoxin was dissolved in DMSO to prepare a solution of 10 mg mL^−1^. All AG samples were prepared into 100 mg mL^−1^ stock solution with distilled water. Ginsenoside Rg3, ginsenoside Rg5, ginsenoside Rg6, malic acid, quinic acid, L-argininosuccinic acid, and pseudoginsenoside F11 were prepared into a stock solution of 40 mg mL^−1^ with DMSO and diluted to the desired concentration before the experiment. AG samples (for UHPLC-MS analysis) were accurately weighed and dissolved in acetonitrile and methanol through a filter membrane to prepare solutions of 5 mg mL^−1^ concentration before the experiment.

### 2.4 Biological activities of the American ginseng samples on the zebrafish

The 48 h of post fertilization (hpf) zebrafish, without the embryonic membrane, were placed in a 6-well plate at the rate of 20 larvae/well and divided into the control (Con), model, positive control (10 μg mL^−1^ digoxin), and AG (100 μg mL^−1^) groups. The positive control group and AG groups were pre-protected for 4 h. Then, all the groups (excluding the control group) were treated with 200 μM verapamil hydrochloride (S. Li et al., 2021) for 1 h to induce heart failure (Figure S1). The heart of zebrafish larvae was then imaged with inverted microscopy (Olympus IX83, Japan) (Supplementary Figure S1). The pericardial area, the venous congestion area (Lu et al., 2020), and the distance between the sinus venosus and bulbus arteriosus (SV–BA) of the heart (Wan et al., 2021) were counted by Image Pro Plus5.1 software. The data were analyzed by one-way analysis of variance (ANOVA) using GraphPad Prism 6.01 software and expressed as the mean ± standard error (SE). In order to evaluate the comprehensive anti-heart failure activity and prevent the incompatibility of statistical indicators, we proposed an integrated index, heart failure improvement rate, for comprehensively evaluating the activity of AG.

### 2.5 Untargeted metabolomics analysis and component identification of American ginseng

In total, 12 batches of AG samples collected from two regions with significantly different bioactivity (seven from Wendeng of Shandong province and five from Xinbin of Liaoning province) were used for untargeted metabolomics modeling analysis. Metabolomics analysis was performed using the Thermo Scientific™ Q Exactive™ Combination Quadrupole Orbitrap™ Mass Spectrometer under the following chromatographic conditions: an ACQUITY UHPLC BEH C18 column (1.7 μm, 2.1 × 100 mm, Waters) was used as the chromatographic column in the positive-ion mode. The column temperature was set to 50°C. The sample injection volume was 10 μL. The mobile phase was composed of water containing 0.1% formic acid (A) and acetonitrile containing 0.1% formic acid (B) at the flow rate of 0.35 ml/min. The gradient elution procedure was performed as follows: 0–1 min, 5% B; 1–24 min, 5–100% B; 24–27.5 min, 100% B; and 27.5–30 min, 5% B. The MS conditions were as follows: electrospray ion sources were used in the positive-ion mode; spray voltage (kV): +3.8; capillary temperature (°C): 320; aux gas heater temperature (°C): 350; sheath gas flow rate (Arb): 35; aux gas flow rate (Arb): 8; S-lens RF level: 50; mass range (m/z): 70–1050; full ms resolution: 70000; MS/MS resolution: 17500; TopN: 5; and NCE/stepped NCE: 20, 40. Except for the spray voltage (kV) -3.0, other conditions were identical in the negative-ion mode.

The raw data of 12 samples and the quality control (QC) samples were processed and converted by Xcalibur 2.2.0 software and ProteoWizard 3.0.21063 software, and then the processed data were uploaded to XCMSonline (Version 3.7.1) for analyses. Mathematical models such as principal component analysis (PCA) and orthogonal partial least squares-discriminant analysis (OPLS-DA) were constructed based on the component information of samples with large activity differences through a smart cloud platform for metabolite identification and biomarker discovery (One-MAP, Dalian ChemDataSolution Information Technology Co., Ltd.). With the VIP (variables important in the projection) value >1.5, *p* < 0.05 and the absolute value of the correlation coefficient |pcorr| >0.58 as the screening conditions, the selected fragment ions were used for qualitative analysis to identify the potential differential components.

### 2.6 Activity validation and mechanism prediction of potential active components

The zebrafish heart failure model was also applied to verify whether those identified components in AG could be used as the pharmacodynamic substances. Accordingly, the heart failure model in zebrafish was basically the same as that described earlier. The zebrafish was divided into control, model, positive control, and ginsenoside Rg3 (25 μg mL^−1^, 50 μg mL^−1^, and 100 μg mL^−1^), ginsenoside Rg5 (0.25 μg mL^−1^, 0.5 μg mL^−1^, and 1 μg mL^−1^), ginsenoside Rg6 (25 μg mL^−1^, 50 μg mL^−1^, and 100 μg mL^−1^), malic acid (10 μg mL^−1^, 25 μg mL^−1^, and 50 μg mL^−1^), quinic acid (25 μg mL^−1^, 50 μg mL^−1^, and 100 μg mL^−1^), L-argininosuccinic acid (25 μg mL^−1^, 50 μg mL^−1^, and 100 μg mL^−1^), and pseudoginsenoside F11 (10 μg mL^−1^, 25 μg mL^−1^, and 50 μg mL^−1^) groups. The concentration-tested groups are mentioned in the Results section.

The targets of the screened active compounds were found on the Traditional Chinese Medicine Systems Pharmacology Database and Analysis Platform (TCMSP, http://tcmspw.com/tcmsp.php). Heart failure-related targets were searched in the GeneCard database (http://www.genecards.org). Their common targets served as core targets. Core targets were imported into the DAVID database (https://david.ncifcrf.gov/) to obtain relevant pathways. The screened active compounds, core targets, and pathways were input into Cytoscape software 3.7.1 to obtain a “compound–target–pathway (C-T-P) network.” The targets with the top degree value in the “C-T-P” network were selected for further molecular docking validation. First, the compounds were structure-optimized with Chem3D software to minimize their energy. The water molecules were then removed from the inactive pocket, and OPLS_2005 force was added into the inactive pocket by Protein Preparation Wizard for energy minimization. Glide’s XP (extra precision) mode was selected for docking. The ligand supplied in the RCSB PDB database (https://www.rcsb.org/) served as the positive control. The position of the original ligand molecule was used as the active site. The size of the docking box and other settings were maintained as default parameters. After docking completion, the 2D mode of docking results was displayed.

### 2.7 Total RNA extraction and quantitative real-time polymerase chain reaction

According to the results of activity verification and target prediction, eight key targets, namely, FGF1, FGF2, VEGFA, STAT3, BCL2L1, MET, PPARA, and CHRNA7 were verified in zebrafish. The groups with the best efficacy of each active component were selected for quantitative real-time polymerase chain reaction (Q-PCR). Zebrafish were divided into the control, model, and drug groups, and 50 zebrafish were included into each group. After removing water, the zebrafish were stored in a -80°C refrigerator for subsequent experiments. The total RNA was extracted using an ultrapure RNA extraction kit (Kangwei Century Biotechnology Co., Ltd. Jiangsu, China) according to the procedure provided by the manufacturer. The RNA concentration was determined and was quantified using an ultramicro-spectrophotometer (Thermo Fisher, United States). The reverse transcription process was performed on ice. The cDNA obtained by the reverse transcription process was directly subjected to Q-PCR analysis. The PCR mixtures contained 2 μL of the cDNA product, template, 10 μM Primer F and Primer R, ROX Reference Dye, and RNase free water in a total volume of 20 μL. Amplification was performed under the following conditions: initial denaturation at 95°C twice for 30 s, 40 cycles of denaturation at 95°C for 15 s, and annealing and extension at 60°C for 30 s three times. All data were determined by the 2^−ΔΔCt^ method and were normalized to the β-actin mRNA signals in each sample. The primer sequences for Q-PCR assay are listed in Table 2.

**TABLE 2:** Primer sequences for Q-PCR assay.

Gene	Forward primer	Reverse primer
FGF1	5'-GCT​CAT​GTC​TGG​TCT​GGC​TT-3'	5'-CAC​ATG​CTT​GAG​GTA​TTT​GGC​A-3’
FGF2	5'-GTA​CCA​ACC​GTT​TCC​TTG​CC-3’	5'-TAC​CAG​TCG​GGA​TAC​TTG​CG-3’
VEGFA	5'-GCT​GTA​ATG​ATG​AGG​CGC​TCG-3’	5'-AAG​GCT​CAC​AGT​GGT​TTT​CTT​TCT-3’
BCL2L1	5'-GGG​CTT​GTT​TGC​TTG​GTT​GA-3’	5'-AGA​ACA​CAG​TGC​ACA​CCC​TT-3’
MET	5'-CGG​TCG​TGT​TGT​GAG​GTT​CT-3’	5'-TGA​AGG​GGC​CAA​ATC​CAT​GT-3’
CHRNRA	5'-CAT​CTC​TAC​TCT​GGC​CCT​GC-3’	5'-CAC​AGA​GTC​AGA​TGT​TGC​CG-3’
STAT3	5'-GCT​TCA​GCA​GAA​GGT​CTC​GT-3’	5'-GAT​GAC​AAG​GGG​TCG​GTC​AG-3’
PPARA	5'-TCA​GGA​CGA​GTT​CAC​CTC​CA-3’	5'-GTC​CGA​CGG​AAG​AAA​CCC​TT-3’
β-Actin	5'-TGC​TCT​GTA​TGG​CGC​ATT​GA-3’	5'-AGG​GGC​CAT​CCA​CAG​TCT​TC-3’

## 3 Results

### 3.1 Evaluation of the anti-heart failure effects of American ginseng samples from various regions

**FIGURE 1:**
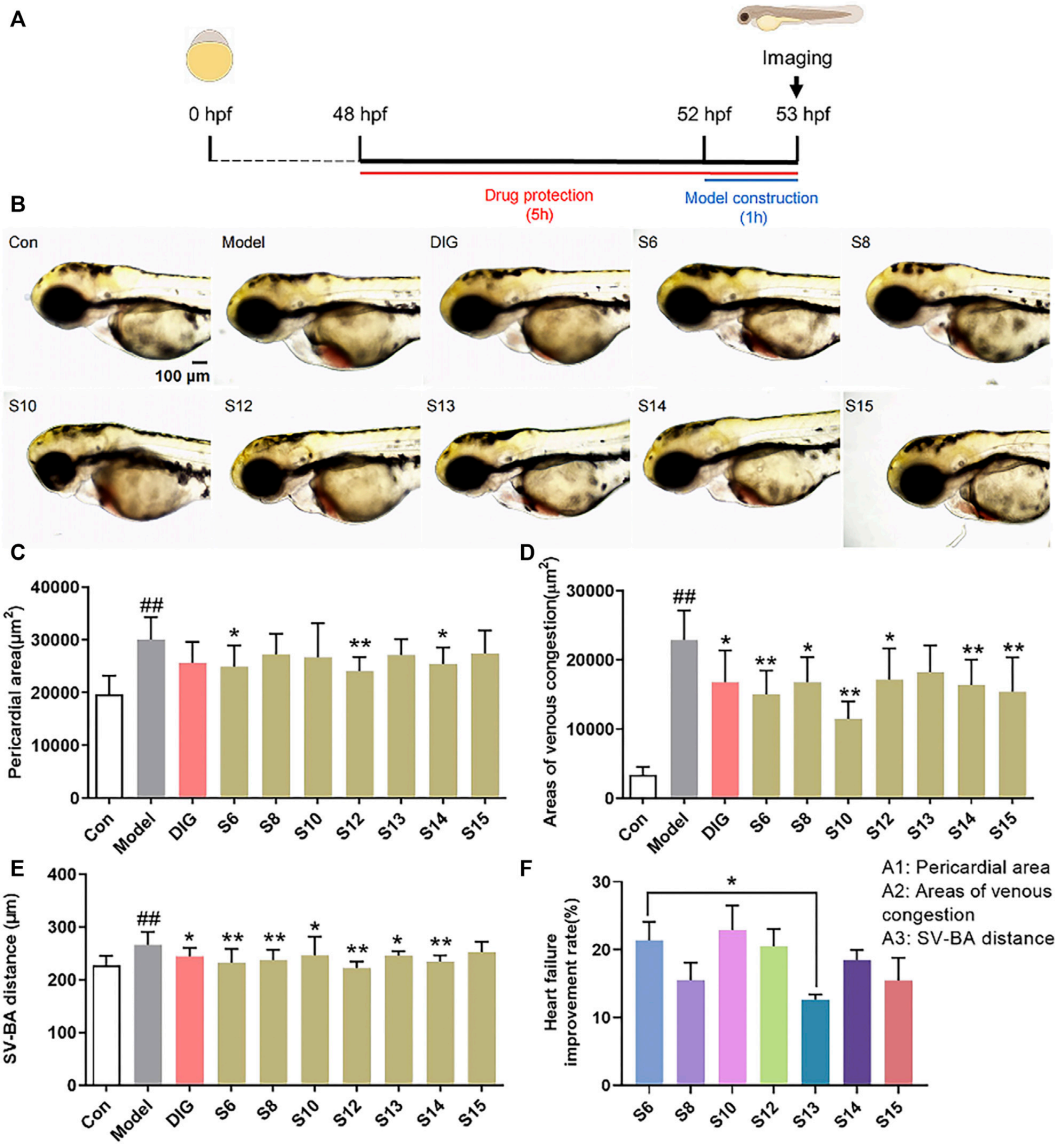
Comparison of the anti-heart failure activities of AG collected from different regions. **(A)** Diagram of drug treatment in the zebrafish heart failure experiment; **(B)** phenotypic micrograph of the zebrafish in the heart; **(C)** statistical results of the pericardial area of the zebrafish heart. *n* = 10; **(D)** statistical results of the venous congestion area of the heart in zebrafish. *n* = 10; **(E)** statistical results of SV–BA in the zebrafish hearts in all groups. *n* = 10; and **(F)** comparison of the improvement rate of heart failure of AG samples from different regions. Heart failure improvement rate = (A1+A2+A3)/3, A = (Φ-a)/Φ*100%, Φ: Mean values of the model groups, **(A)** values of sample groups; ##*p* < 0.01 vs. the control group; *p < 0.05 and **p < 0.01 vs. the model group.

The pericardial area, venous congestion area, and SV–BA distance of the zebrafish heart in the model group were significantly increased compared with those of the control group (*p* < 0.01), indicating the successful induction of heart failure in the zebrafish by verapamil hydrochloride treatment. The venous congestion area and SV–BA distance of the zebrafish heart were remarkably reversed after treatment with the positive drug digoxin (*p* < 0.05). Pericardial areas in the zebrafish were significantly reduced in the S6, S12, and S14 treatment groups than in the model group (Figure 1C). However, the pericardial area of the zebrafish in the S8, S13, and S15 treatment groups did not show a difference compared with that of the zebrafish in the model group. After the treatment, venous congestion in the zebrafish was relieved in the S6, S8, S10, S12, S14, and S15 treatment groups compared with the model group (Figure 1D). Our results showed that all AG samples, except S15, markedly decreased the SV–BA distance of the zebrafish heart in their respective groups than in the model group (Figure 1E). As shown in Figure 1F, the improvement rate of S13 for heart failure was the lowest, and the improvement rate of S6 and S10 was higher.

### 3.2 Screening of potential active components from American ginseng using untargeted metabolomics

**FIGURE 2:**
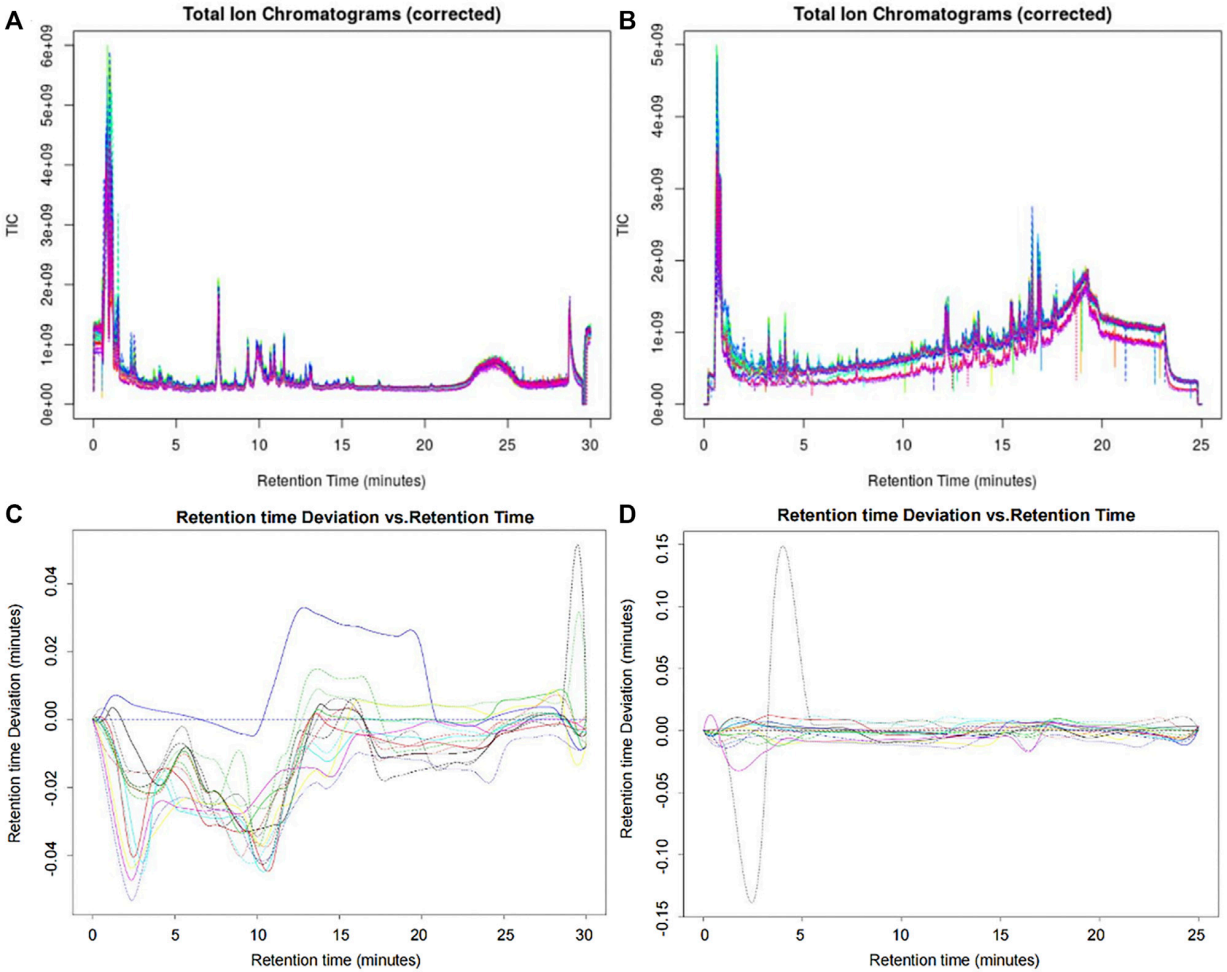
Superposition map of the total ion chromatograms of AG samples. **(A)** Corrected total ion chromatogram in the positive ion mode; **(B)** corrected total ion chromatogram in the negative ion mode; **(C)** retention time deviation diagram of the AG samples in the positive ion mode; and **(D)** retention time deviation diagram of the AG samples in the negative ion mode.

The original data were preprocessed to obtain the superposition map of total ion chromatograms (Figures 2A,B). The calibration process was visualized using the calibration curve of retention time. Furthermore, the retention time of the peak should be within the ± 0.2-min retention time window of the retention time observed in the available standard. As shown in Figures 2C,D, the peak of the AG sample met the standard. After preprocessing using XCMSonline software, a total of 4535 chromatographic peaks with *p* < 0.01 in the positive ion mode and 2668 chromatographic peaks in the negative ion mode were obtained.

**FIGURE 3:**
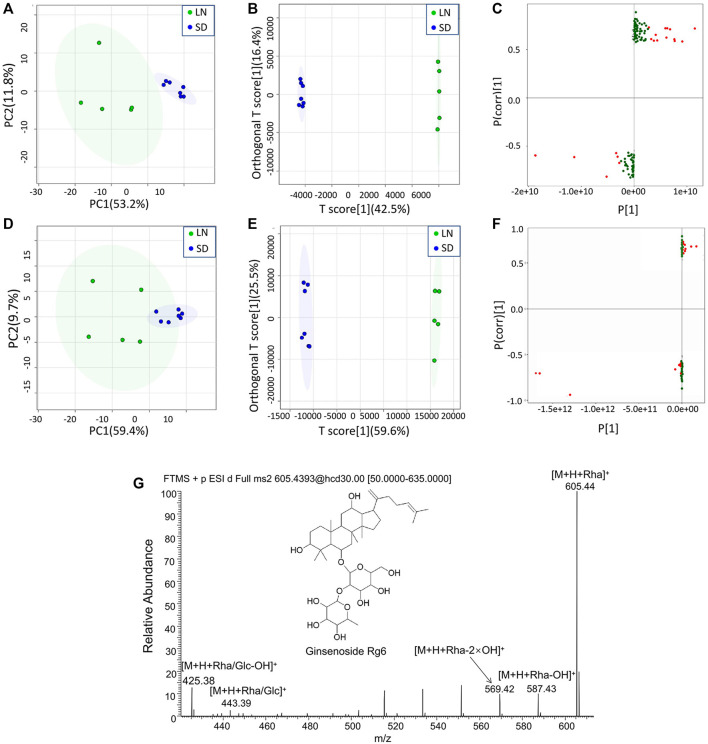
Multivariate statistical analyses of the metabolomic data of GF. **(A)** PCA scatter plot in the positive ion mode; **(B)** OPLA-DA scatter plot in the positive ion mode; **(C)** S-plots for the OPLA-DA in the positive ion mode; **(D)** PCA scatter plot in the negative ion mode; **(E)** OPLA-DA scatter plot in the negative ion mode; **(F)** S-plots for the OPLA-DA in the negative ion mode; and **(G)** MS2 spectra of ginsenoside Rg6.

The MS data of 12 batches of AG were inputted into OneMap for principal component analysis. As shown in Figure 3A, the PCA scatter plot was constructed by the first principal component (53.2%) and the second principal component (11.8%) in the positive ion mode. It is observed that the AG samples obtained from two regions were clearly separated, indicating that some compounds of the two groups were different. The first and second principal components were 53.1% and 15.3% in the negative ion mode, respectively (Figure 3D). The AG samples were not completely separated from the scatter plot, and thus further modeling and analysis were required.

After the principal component analysis, the supervised OPLS-DA was used to further analyze the differences between these two groups. As shown in Figures 3B, E OPLS-DA analysis could significantly distinguish the AG samples from two regions. The values of R2Y (cum) and Q2 (cum) in the positive and negative ion models were 0.98 and 0.85 and 0.92, and 0.84, respectively, indicating the good measurability and applicability of the OPLS-DA model. The permutation test validated whether the OPLS-DA model was overfitted. The results of the PT were R2 = (0.0, 0.21) and Q2 = (0.0, -0.51) and R2 = (0.0, 0.33) and Q2 = (0.0, -0.65). The intersection of the regression line of the Q2 value with the ordinate was less than zero, and all Q2 values on the left were less than the highest value on the right, indicating the usability of the model. VIP value > 1.5, *p* < 0.05, and |pcorr| > 0.58 were used to screen the potentially important fragment ions (Table 3). The results were represented using S-plots of the OPLA-DA model (Figures 3C,F). The protective effects of a total of nine potentially important components against heart failure were identified by standard substances or MS/MS data (Table 4). Compound 3 was an example of displaying the structure identification process of the compounds. Compound 3 gaged a quasi-molecular ion of *m/z* 767.4917[M + H]^+^; therefore, the calculated molecular formula was deduced as C_42_H_70_O_12_ with an error of 3.7 ppm, which was consistent with that of ginsenoside Rg6. The MS/MS showed that obvious *m/z* 605.4393, 587.4288, 569.4183, 443.3870, and 425.3762 fragment ions were obtained compared with the literature data, and the fragment ion was deduced as *m/z* 605.4393 [M + H-Rha]^+^; 587.4288 [M + H-Rha-OH]^+^; 569.4183 [M + H-Rha-2OH]^+^; 443.3870 [M + H-Rha/Glc]^+^; and 425.3762 [M + H-Rha/Glc-OH]^+^. Accordingly, compound 3 was speculated as ginsenoside Rg6 (Figure 3G).

**TABLE 3:** Screening of the potentially active components of AG against heart failure.

VarID	*P* (T-test)	VIP	Ion mode
V20188	0.023302	1.56008179	neg
V20301	0.014374	1.648690526	neg
V33038	0.0030027	1.81109426	pos
V18051	0.031844	1.526483347	neg
V11178	0.033366	1.625269504	neg
V6096	0.0083365	1.741859991	neg
V2878	0.028691	1.515140861	neg
V15698	0.0010554	1.922905341	pos
V1259	0.010402	1.696352652	neg

**TABLE 4:** Identification of differential active components from AG using the UHPLC-QE-Orbitrap-MS.

NO.	Rt	Measured m/z	Calculated m/z	ppm	MSE fragment ions	Compound identification	Chemical structure
1	18.19	765.4783 [M-H]-	765.4789	−0.74	811.4829 [M + HCOO]-	Ginsenoside Rg5^a^	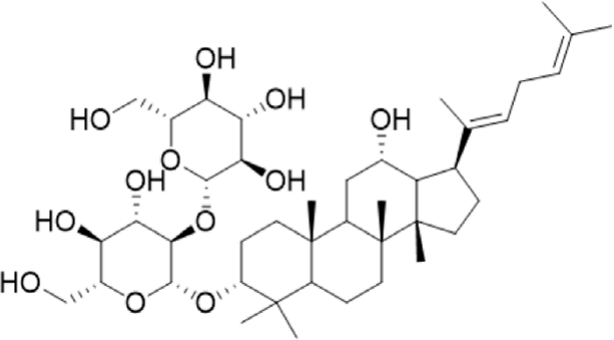
2	17.63	783.4889 [M-H]-	783.4895	−0.71	783.4895653.4199[M-H-Rha-H2O]-	Ginsenoside Rg3^b^	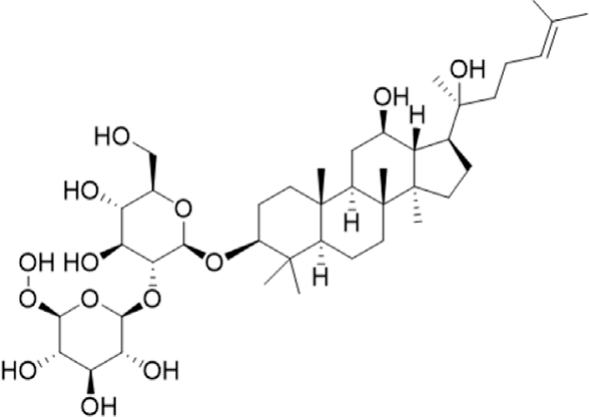
3	14.55	767.4917[M + H]+	767.4946	−3.7	605.4393 [M + H-Rha]+; 587.4288[M + H-Rha-OH]+; 569.4183 [M + H-Rha-2OH]+; 443.3870 [M + H-Rha/Glc]+; 425.3762 [M + H-Rha/Glc-OH]+	Ginsenoside Rg6^b^	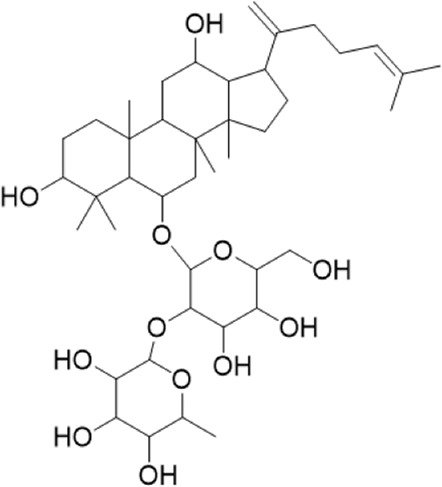
4	10.72	799.4831 [M-H]-	799.4829	2.3	653.4199[M-H-Rha-H2O]-	Pseudoginsenoside F11^b^	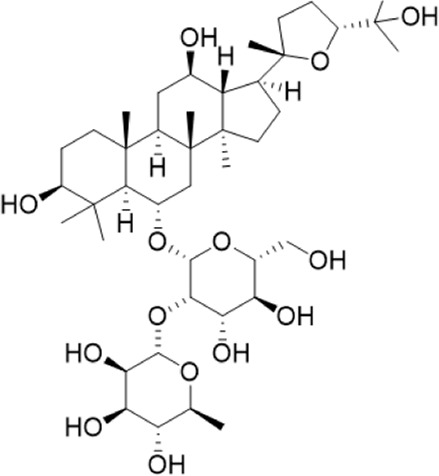
5	3.68	191.0557[M-H]-	191.0556	0.58	145.0726[M + HCOO]-	Quinic acid^b^	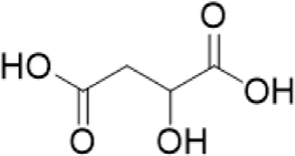
6	0.62	133.0139 [M-H]-	133.0137	1.7	115.0325[M-H-H2O]^-^	Malic acid^b^	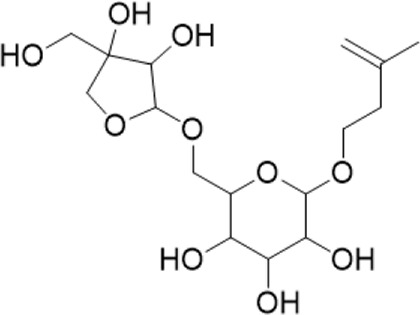
7	6.69	379.1602[M-H]-	379.1604	−0.64	361.1384[M-H-H2O]-	3-methyl-3-butenyl-apinosyl (1→6) glucoside^b^	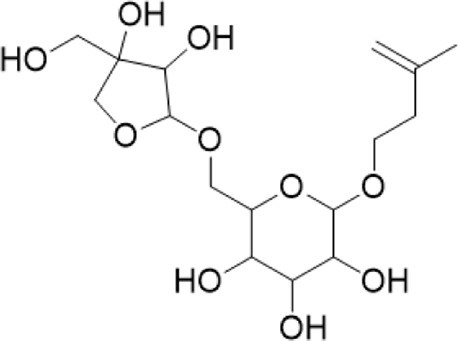
8	0.77	291.1292 [M + H]+	291.1305	−4.4		L-argininosuccinic acid^b^	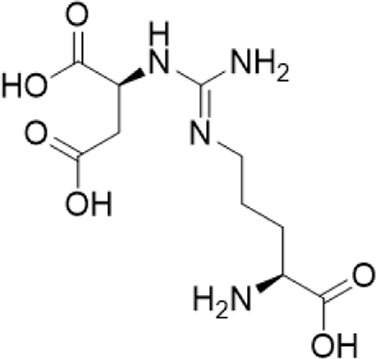
9	4.86	264.1013722[M-H]-	264.1025	−42		Annonaine^b^	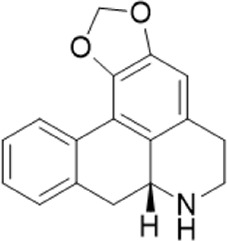

“a” refer to an identified component by standard substances; “b” refers to a speculated component; identification of active components according to MS data from reference (Huang et al., 2019; Y. Xia et al., 2018; Chen et al., 2017).

### 3.3 Bioactivity evaluation of the identified bioactive compounds by the zebrafish model

**FIGURE 4:**
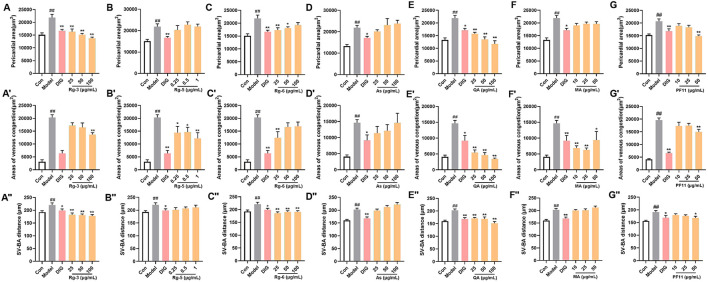
Validation of the anti-heart failure activity of ginsenoside Rg3, ginsenoside Rg5, ginsenoside Rg6, malic acid, L-argininosuccinic acid, quinic acid, and pseudoginsenoside F11. **(A–G)** Statistical results of the pericardial area of the zebrafish. *n* = 10; **(A’–G’)** statistical results of the venous congestion area of the heart in zebrafish. *n* = 10; **(A**”**–G**”**)** statistical results of the SV**–**BA distance in zebrafish hearts. *n* = 10. ##*p* < 0.01 vs. the control group,*p < 0.05 and **p < 0.01 vs. the model group.

Potential differential components screened by metabolomics were validated using the zebrafish model. Among them, ginsenoside Rg3, ginsenoside Rg5, ginsenoside Rg6, malic acid, quinic acid, and pseudoginsenoside F11 could significantly improve heart failure caused by verapamil hydrochloride treatment in zebrafish. Ginsenoside Rg3, ginsenoside Rg6, quinic acid, and pseudoginsenoside F11 could improve the expansion of the pericardial area, venous congestion area, and SV–BA distance in zebrafish hearts. Ginsenoside Rg5 and malic acid only ameliorated venous congestion in zebrafish hearts. L-argininosuccinic acid with different concentrations did not improve heart failure in zebrafish (Figure 4).

### 3.4 Network prediction of the action mechanism of bioactive compounds

**FIGURE 5:**
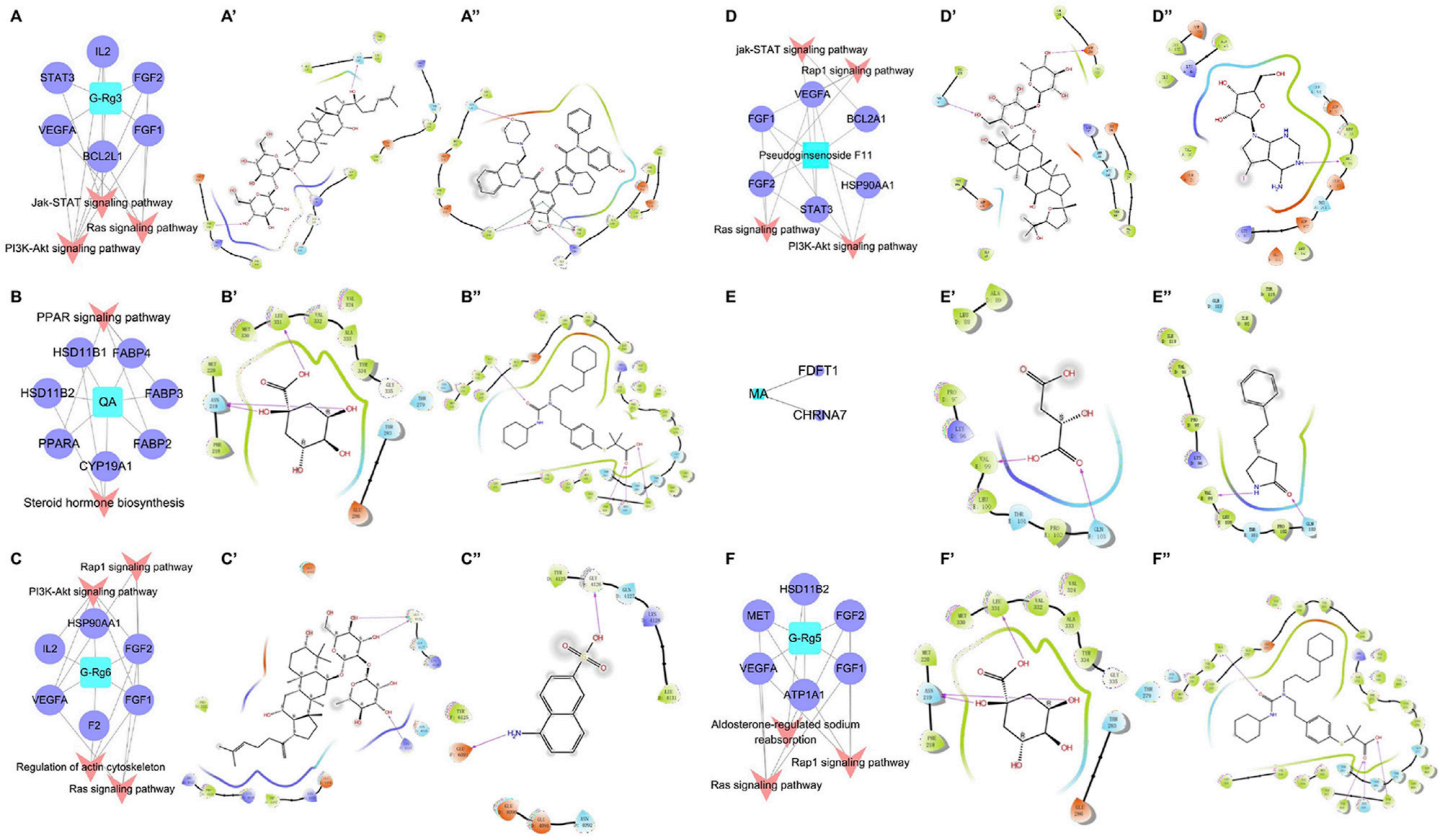
C-T-P network of the anti-heart failure and molecular docking 2D diagram. **(A–F)** C-T-P network of ginsenoside Rg3, quinic acid, ginsenoside Rg6, pseudoginsenoside F11, malic acid, and ginsenoside Rg5; **(A’–F’)** molecular docking 2D diagram of the bioactive molecules; **(A”–F”)** molecular docking 2D diagram of positive molecules.

The targets and regulatory pathways of ginsenoside Rg3, ginsenoside Rg5, ginsenoside Rg6, malic acid, quinic acid, and pseudoginsenoside F11 are shown in Figure 5A–5F. The results showed that these six compounds may, respectively, target alpha 7 nicotinic acetylcholine receptor (CHRNA7), peroxisome proliferator-activated receptor alpha (PPARA), B-cell lymphoma-2 like 1 (BCL2L1), hepatocyte growth factor receptor (MET), fibroblast growth factor 1 (FGF1), and signal transducer and activator of transcription 3 (STAT3) involved in multiple signaling pathways. Moreover, the results showed high binding energy in molecular docking. Malic acid targeted two targets, namely, farnesyl diphosphate farnesyl transferase 1 (FDFT1) and CHRNA7. Quinic acid targeted two pathways and seven targets, including PPARA, fatty acid-binding protein 2 (FABP2), and FABP3. In the network prediction analysis, ginsenoside Rg3 targeted BCL2L1, which was the key target in the signaling pathways. MET encoded a receptor tyrosine kinase c-MET for a hepatocyte growth factor (HGF) (Mo and Liu, 2017), which was an important target of ginsenoside Rg5 against heart failure. Ginsenoside Rg6 targeted six targets and four pathways, as performed by pseudoginsenoside F11.

The information on the predicted targets and related molecular docking is shown in Table 5. The action mode of the six pharmacodynamic compounds and their corresponding docking targets is displayed (Figure 5A’∼5F’ and 5A”∼ 5F”). The binding energy of malic acid (-4.117 kcal/mol) was lower than that of the positive control molecule attached to the receptor. The carboxyl group formed hydrogen bonds with two amino acid residues in the active pockets of Val99 and GLN103. The binding energy of quinic acid to the PPARA target was -7.835 kcal/mol. Although the binding energy of quinic acid was lower than that of the molecule attached to the receptor, the binding energy of quinic acid was high as it involved the formation of three hydrogen bonds between the hydroxyl groups and two amino acid residues in the active pocket of ASN219 and LEU331. The binding energy of ginsenoside Rg3 was slightly different from that of the positive control molecule attached to the receptor. The hydroxyl groups at carbon 20 and the glycoside part of ginsenoside Rg3 formed three hydrogen bonds with three amino acid residues (TYR108, ARG146, and GLN118) of the active pocket. Ginsenoside Rg5 formed five hydrogen bonds between the four amino-acid residues (ASP1164, GLU1082, LYS1161, and TYR1159) in the active pocket and the hydroxyl group on the glucose ring structure in the polar glycoside part. The binding energy of ginsenoside Rg6 was -5.438 kcal/mol, which was higher than that of the receptor FGF1 ligand. Ginsenoside Rg6 mainly formed three hydrogen bonds between the two amino acid residues (GLY4126 and LYS4113) in the active pocket and the hydroxyl groups on the glycoside in the polar part. Pseudoginsenoside F11 formed two hydrogen bonds with the amino acid residues.

**TABLE 5:** Information of the compounds docking proteins.

Compound	Target	PDB ID
Malic acid(MA)	CHRNA7	5AFN
Quinic acid(QA)	PPARA	6KB3
Ginsenoside Rg3(G-Rg3)	BCL2L1	6GL8
Ginsenoside Rg5(G-Rg5)	MET	4R1V
Ginsenoside Rg6(G-Rg6)	FGF1	1HKN
Pseudoginsenoside F11 (PF11)	STAT3	5AX3

### 3.5 Anti-heart failure-related gene expression of key compounds in American ginseng

**FIGURE 6:**
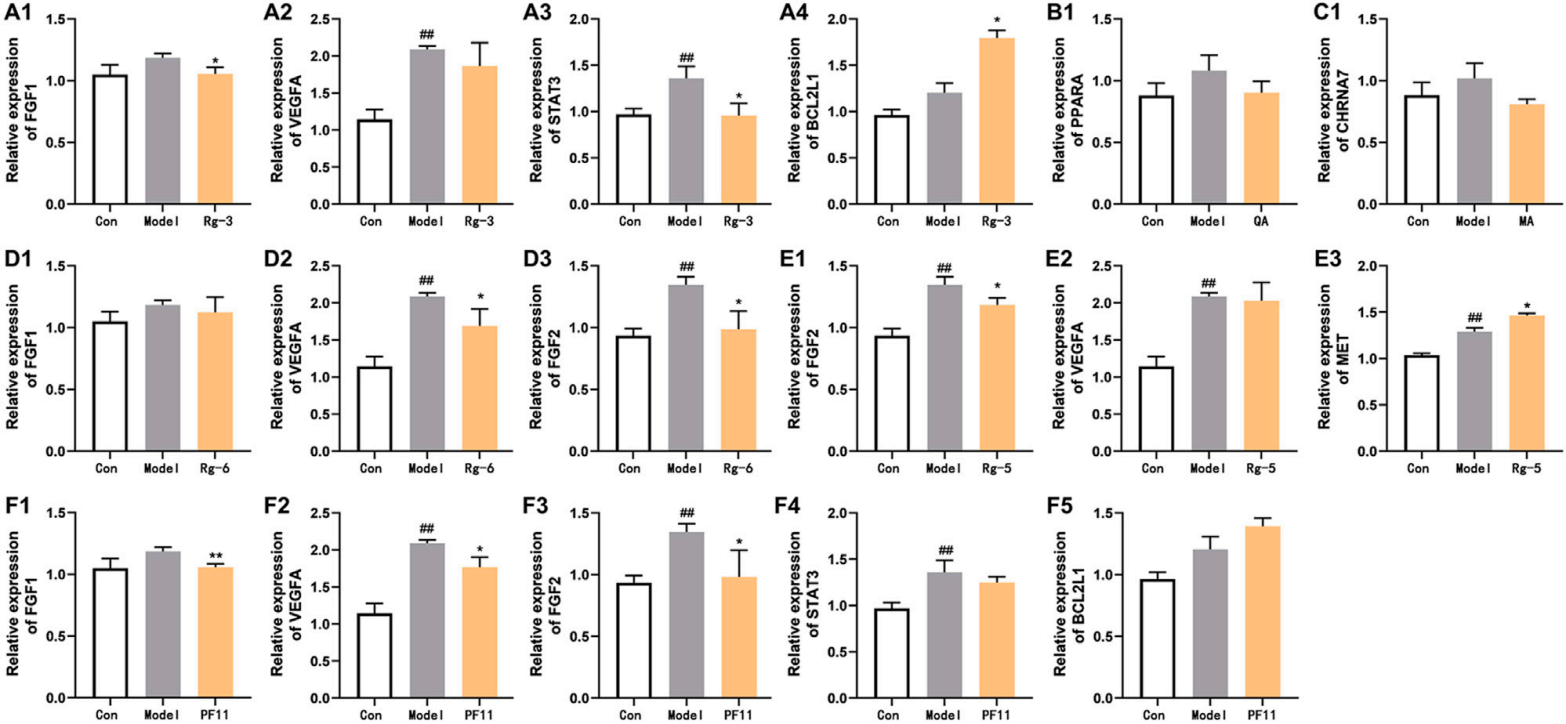
Effects of potential active compounds on heart failure-related gene expression. **(A–F)** Results of gene expression in zebrafish treated with ginsenoside Rg3, quinic acid, malic acid, ginsenoside Rg6, ginsenoside Rg5, and pseudoginsenoside F11, ##*p* < 0.01 vs. the control group,*p < 0.05 and **p < 0.01 vs. the model group.

To elucidate the mechanisms underlying the amelioration effect of key active compounds on heart failure in the zebrafish, the mRNA levels of genes involved in the regulation of heart failure were determined by Q-PCR analysis (Figure 6). In the verapamil-treated group, the mRNA levels of FGF2, VEGFA, and STAT3 were significantly increased (*p* < 0.01) compared with those of the control group. The mRNA expression of FGF1, PPARA, and CHRNA7 in the model group was moderately elevated compared with that of the control group. Treatment with ginsenoside Rg3 inhibited the mRNA expression levels of both FGF1 and STAT3 (*p* < 0.05). Ginsenoside Rg5 treatment significantly decreased the mRNA expression levels of FGF2 compared with the verapamil-treated zebrafish (*p* < 0.05), and the mRNA expression levels of VEGFA were not statistically significant in comparison with the model group. Ginsenoside Rg6 treatment obviously attenuated the mRNA expression levels of FGF2 and VEGFA in the model group (*p* < 0.05). The mRNA expression levels of FGF1, VEGFA, and FGF2 in the pseudoginsenosideF11-treated group were significantly lower than those in the model group (*p* < 0.05). Quinic acid and malic acid had no significant effect on the mRNA expression of PPARA and CHRNA7, but both these two components reduced the mRNA expression levels of PPARA and CHRNA7, respectively.

## 4 Discussion

As a traditional Chinese medicine for replenishing energy, American ginseng was reported to have a protective effect on the heart (L. Yang et al., 2020). Therefore, a zebrafish heart failure model was established as a tool to evaluate the main activities of AG in this study. As a new vertebrate model organism between cells and mammals, zebrafish has been widely used in the screening of cardioprotective drugs in recent years (Da Silveira Cavalcante and Tessier, 2021). In this study, zebrafish was treated with verapamil hydrochloride, a calcium ion blocker, and established a fast drug screening model for heart failure. AG is widely distributed in low mountains near 37° north latitude, such as the Great Lakes region of North America, Northeast China, and North China. There are currently four producing areas in China: Northeast China, Shaanxi, Beijing, and Shandong. Studies had shown that the growth of AG was affected by climate, temperature, water, soil, and other environmental factors (J. Liang et al., 2019). Our study found that the anti-heart failure activities of AG in these four producing areas were different due to different growth environments.

Various case studies have reported that many components in AG had protective effects on the heart. Currently, the identified ginsenosides are the main secondary metabolites of *Panax* L. plants (W. Fan et al., 2020). Ginsenoside Re improves isoproterenol-induced heart failure in rats (Wang Q. W. et al., 2019). Ginsenoside Rh2 improves cardiac fibrosis in type 1-like diabetic rats (Lo et al., 2017). Ginsenoside Rb1 attenuates myocardial ischemia/reperfusion injury through inhibiting ROS production from mitochondrial complex I (L. Jiang et al., 2021). In this study, ginsenoside Rg3, ginsenoside Rg5, ginsenoside Rg6, malic acid, quinic acid, and pseudoginsenoside F11 were screened as the P-markers of AG. Of note, the anti-heart failure activity of ginsenoside Rg6, pseudoginsenoside F11, and quinic acid has been reported for the first time.

Pharmacodynamic marker (P-marker) is a new concept proposed on the basis of quality marker (Q-marker) of TCM. P-markers are the key components of TCM to exert some or all of pharmacodynamic effects. The difference between the P-markers and Q-markers is that the latter are all markers reflecting the quality of TCM, while the former focuses on markers directly related to bioactive efficacy. This study integrated the advantages of the zebrafish model and metabolomics and established a new model of “component-activity” integrated research strategy, which can quickly discover markers related to specific activities. This strategy has been applied in the research on the pharmacodynamic components of Gardenia Fructus in our previous research (Y. P. Shi et al., 2020).

Network pharmacology is an emerging discipline integrating multi-directional pharmacology, bioinformatics, and physical chemistry. The emergence of network pharmacology provides a new method for multi-component and multi-target research of traditional Chinese medicine (Qu et al., 2021). Network pharmacology technology can realize multi-component target identification and can predict the target for known or new drug components. The pharmacodynamic components found in this study are highly correlated with FGF1, FGF2, VEGFA, STAT3, BCL2L1, and other targets, and the prediction of network pharmacology provides a basis for subsequent in-depth verification.

The results of the phenotype and gene expression of zebrafish can partially reveal the potential mechanism of action of the P-markers discovered in this study. FGF1, FGF2 (Zhao et al., 2011), and VEGFA (Braile et al., 2020) are considered to be important factors in stimulating endothelial cell proliferation and in promoting angiogenesis. In addition, the structural activation of STAT3 can also promote angiogenesis (Hilfiker-Kleiner et al., 2005). In the current study, the increase of FGF1, FGF2, VEGFA, and STAT3 mRNA expression in verapamil-treated zebrafish suggested that they were likely response to initiate angiogenesis in cardiac injury. After pre-protection with ginsenoside Rg3, ginsenoside Rg5, ginsenoside Rg6, and pseudoginsenoside F11, the cardiac damage of zebrafish was alleviated, speculating that these components may protect the heart by participating in the regulation of cardiac angiogenesis and repairing itself in early-stage heart failure. PPARA is a key regulator of glucose transshipment. Heart failure activates PPARA to regulate fatty acid metabolism and provide energy for cardiomyocytes (Kaimoto et al., 2017). After pre-protection in zebrafish by quinic acid, low mRNA expression of PPARA was observed, suggesting that PPARA activation during heart failure improved myocardial function and energetics. Our data suggested that quinic acid against heart failure may be associated with regulating fatty acid metabolism to supply energy for cardiomyocytes. As a key regulator of apoptosis in the mitochondrial pathway, BCL2L1 is the common anti-apoptotic protein that promotes cell survival (Loo et al., 2020). MET is the tyrosine kinase receptor for HGF, which mainly activates prosurvival pathways, including prevention from apoptosis. The mRNA expression of BCL2L1 and MET was increased after ginsenoside Rg3, ginsenoside Rg5, and pseudoginsenoside F11 treatment, indicating that these three components may protect cardiomyocytes from damage by inhibiting apoptosis to improve heart failure. Acetylcholine binding to the CHRNA7 expressed on macrophages polarizes the pro-inflammatory into anti-inflammatory subtypes (Chung et al., 2020). STAT3 has an anti-inflammatory effect by restraining pro-inflammatory gene transcription, initiating efficient reparative mechanisms (Hillmer et al., 2016). After pre-protection by malic acid, ginsenoside Rg3, and pseudoginsenoside F11, the expressions of CHRNA7 and STAT3 mRNA were reduced, indicating that heart protection of malic acid, ginsenoside Rg3, and pseudoginsenoside F11 may be related with the activation of inflammatory response.

## 5 Conclusion

To summarize, six key P-markers of anti-heart failure in AG were identified using the zebrafish model and metabolomics technology. This study provided a new strategy for the discovery and identification of potentially active components of TCM, which had advantages over traditional research patterns. These results might contribute to evaluating the interior quality of AG.

## Data Availability

The original contributions presented in the study are included in the article/Supplementary Material; further inquiries can be directed to the corresponding authors.
