# Supplementary material for: Identification of key pharmacodynamic markers of American ginseng against heart failure based on metabolomics and zebrafish model
Source: Front Pharmacol. 2022 Oct 14;13:909084. doi: 10.3389/fphar.2022.909084 (PMC9614665; doi:10.3389/fphar.2022.909084)
Supplement: Supplementary file 1 [file DataSheet1.docx]

Supplementary Material

### **Construction of the heart failure model**

The 48 hours of post fertilization (hpf) zebrafish embryo were treated with pronase E and the larvae were placed in 6-well plates with 30 larvae per well. The grouping is as follows: the control group (Con) , 200 μmol/L(μM), 220μM, 240μM, 260μM verapamil hydrochloride group (treatment for 30 min) and 200μM verapamil hydrochloride group (treatment for 60 min). All groups (excluding the control group) were given verafamil hydrochloride at different concentrations to induce heart failure in zebrafish. The heart of zebrafish larvae were imaged by inverted microscope (Olympus IX83, Japan). The pericardial area and venous congestion area of zebrafish were counted by Image Pro Plus5.1 software.

## **Establishment of heart failure models on zebrafish**

In the present study, the heart failure model was established on zebrafish by the treatment of verapamil hydrochloride (Fig.1A). As shown in Fig.1B, there was no significant difference observed in the pericardial area of zebrafish between the control group and groups treated with verapamil hydrochloride at concentration of 200 μM, 220 μM, 240 μM and 260 μM for 30 min. When zebrafish were treated with verapamil hydrochloride at a concentration of 220 μM for 60 min, the pericardial area was significantly expanded compared with the control group (*P* < 0.01). Treatment with 200 μM and 220 μM verapamil hydrochloride for 30 min did not cause venous congestion in zebrafish (Fig.1C). The venous congestion area was apparently occurred after the treatment with 240 μM and 260 μM verapamil hydrochloride (*P* < 0.05). Venous congestion was obviously induced in zebrafish treated with 200 μM verapamil hydrochloride for 60 min (*P* < 0.01). Therefore, heart failure model in zebrafish was constructed by the treatment of 200 μM verapamil hydrochloride for 60 min.


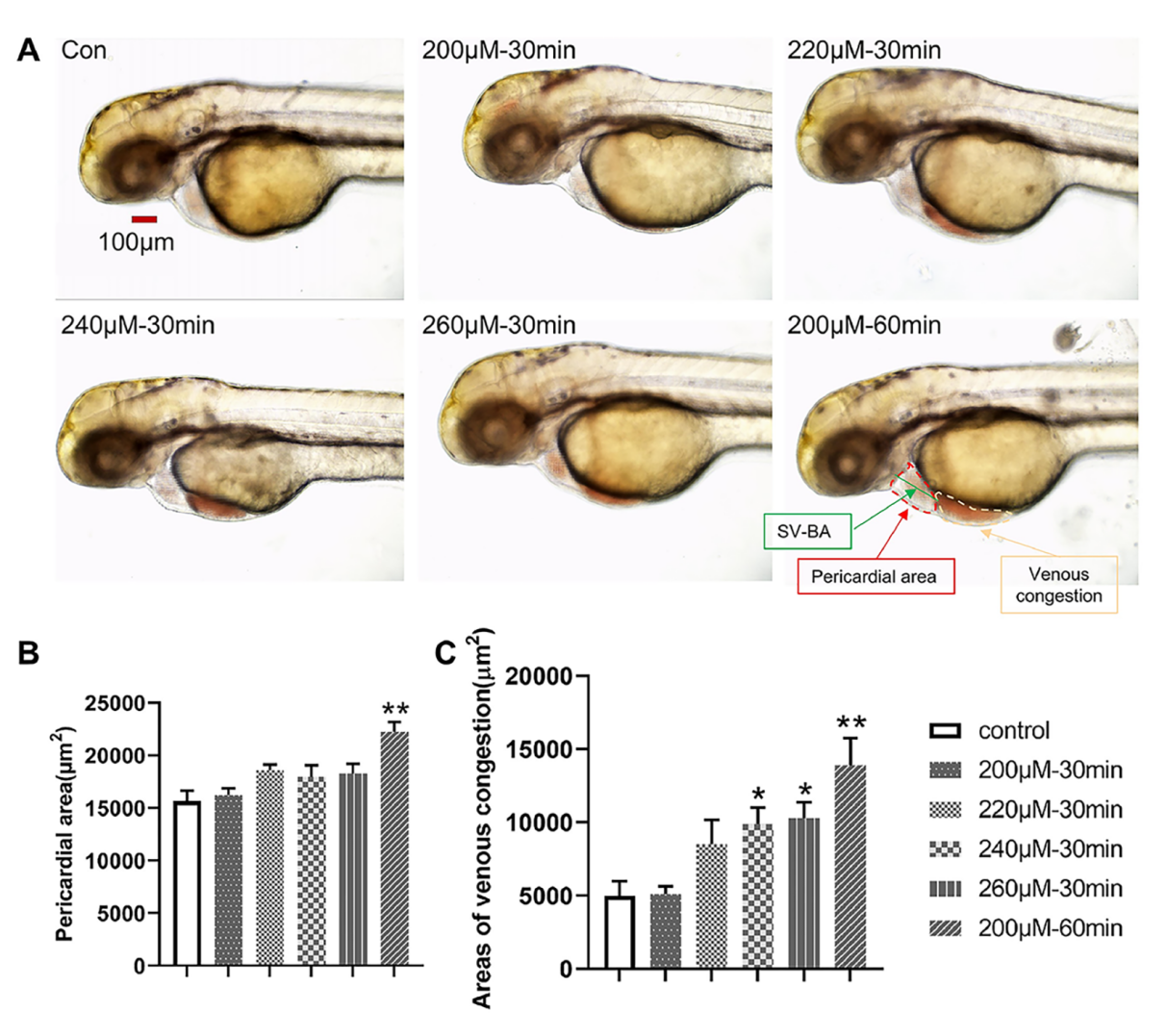


**Fig. S1** Establishment of zebrafish heart failure models. (A) Phenotypic micrograph of the zebrafish heart; (B) The pericardial area of the zebrafish in all groups. n = 10; (C) The venous congestion area of the zebrafish in all groups. n = 10. “*”indicates *P* < 0.05 vs. the control group; “**” indicates *P* < 0.01 vs. the control group.
